# Combining Non-invasive Ventilation with timed position change in the Emergency Department to improve oxygenation and outcomes in patients with COVID-19: A prospective analysis from a low resource setup

**DOI:** 10.12669/pjms.38.ICON-2022.5772

**Published:** 2022-01

**Authors:** Saima Ali, Adeel Khatri, Nida Ghouri, Sama Mukhtar, Suha Zawawi, Syed Ghazanfar Saleem

**Affiliations:** 1 Dr. Saima Ali, FCPS, Emergency Department, Indus Hospital and Health Network (IHHN), Korangi Crossing, Karachi, Pakistan; 2 Dr. Adeel Khatri, FCPS, Emergency Department, Indus Hospital and Health Network (IHHN), Korangi Crossing, Karachi, Pakistan; 3 Ms. Nida Ghouri, M.Sc, Indus Hospital Research Center (IHRC), Indus Hospital and Health Network (IHHN), Korangi Crossing, Karachi, Pakistan; 4 Dr. Sama Mukhtar, FCPS, Emergency Department, Indus Hospital and Health Network (IHHN), Korangi Crossing, Karachi, Pakistan; 5 Dr. Suha Zawawi, MBBS, Emergency Department, Indus Hospital and Health Network (IHHN), Korangi Crossing, Karachi, Pakistan; 6 Dr. Syed Ghazanfar Saleem, FCPS, Indus Hospital and Health Network (IHHN), Korangi Crossing, Karachi, Pakistan

**Keywords:** COVID-19, Non-Invasive Ventilation, Supine Position, Prone Position, ARDS

## Abstract

**Background:**

Moving away from invasive ventilation towards timed position change and non-invasive ventilation is especially of benefit in low and middle income countries, where judicious use of the available healthcare resources is the need of the day. Our study was conducted prospectively to develop strategies for non-invasive ventilation in combination with timed position change of patients to see its impact on their outcome.

**Objectives:**

Non-invasive ventilation has proven to be of benefit in COVID-19 related acute lung injury. The objective of this prospective, cross sectional study was to develop a protocol for the use of non-invasive ventilation with timed position change to improve COVID-19 patients’ outcomes in the Emergency Department (ED).

**Methods:**

All patients presenting with confirmed or suspected COVID-19 were enrolled in the study from March 2020 to October 2020. Data was collected to see the effect of timed position change and non-invasive ventilation on these patients and its effect on delaying or avoiding invasive ventilation.

**Results:**

Of the 207 COVID-19 patients presenting to the IHHN ED, 109(52.7%) had oxygen saturation in the nineties in supine position followed by right lateral in 37(17.9%), sitting up in 30(14.5%), left lateral in 29(14%) and prone position in 2(1%). Maximal oxygenation was achieved with non rebreather mask (NRM) and nasal prongs in 87(42%) of the patients, followed by the use of continuous positive airway pressure (CPAP) in 29(14%).

**Conclusion:**

Most of the patients preferred to stay in the supine position and described it as the position of comfort. When used in combination supine position, patients on NRM with nasal prongs and on CPAP, had oxygen saturation in the nineties. Central obesity was found to be the prime reason for the inability to prone our patients. This needs to be followed up in the current fourth wave of COVID-19 to see the effectiveness of the said modalities.

## INTRODUCTION

With the COVID-19 pandemic now in its fourth wave, critically ill patients are coming to EDs with hypoxia, bilateral lung injury and post COVID sequelae. Since its outbreak in November 2019, many methods for improving oxygenation and patient outcome have been documented in patients with COVID. Most of these were initially focused on early intubation and ventilation and led to a gross overburden of the health-care system in terms of human resource and availability of intensive care unit (ICU) beds and mechanical ventilators.^1^ This led to research to delay or forego invasive ventilation and improve oxygenation through modalities of timed position change and non-invasive ventilation in patients with COVID-19.

Classically, hypoxemic patients with respiratory distress are put in a supine or upright position. Prone position during invasive ventilation has been described in literature as a successful method to increase alveolar recruitment in patients with Acute Respiratory Distress Syndrome (ARDS).^2^ COVID-19 patients are postulated to rapidly progress to ARDS with the observation that prone positioning can improve oxygenation even in non-intubated, spontaneously breathing patients.^3^ However, as the pandemic has progressed, there are reports that intermittent and timed position change of patients to keep them comfortable and effectively oxygenated, can be an alternative approach, as prone position can be difficult to achieve in certain patients.^4^,^5^

In Low-Middle Income Countries (LMICs) like Pakistan, the scarcity of available critical care resources has added insult to injury. To date, the total number of COVID-19 cases in Pakistan have been 1.09 million with the largest burden of disease in the province of Sindh with 406,000 active cases.^6^ With the rationale of minimizing invasive ventilation and judicious utilization of available resources through assessing the effect of timed position change with non-invasive ventilation modalities, a study was conducted on COVID-19 patients presenting to our ED, located in one of the most crowded vicinities in Karachi, the eleventh most populous city in the world.^7^ The idea was to develop pathways that work best for our population during the first wave so that the same can be applied in subsequent waves with wise resource allocation.

The primary objective of this study was to see the effect of improvement of oxygenation by following the position changing protocol in suspected or positive COVID-19 patients with hypoxemia and respiratory distress. The secondary objective was to see the best combination of position change and non-invasive ventilation modalities like nasal cannula, non-re breather mask and CPAP in improving the oxygenation of patients. A follow-up of this study with the use of position preferred by patients and non-invasive ventilation is underway in the current fourth wave.

## METHODS

A prospective, cross sectional study was conducted to see the effects of timed position change and non-invasive ventilation modalities in patients with suspected or diagnosed COVID-19, who came to our ED at The Indus Hospital and Health Network (IHHN), Karachi from March 2020 to October 2020.

The target population was suspected or diagnosed COVID-19 patients who had hypoxemic respiratory failure with high work of breathing. All patients above eighteen years, conscious and awake, spontaneously breathing with a respiratory rate of > 24/minutes and on supplemental oxygen were included in the study. All the patients who were already intubated, had immediate need for intubation, were hemodynamically unstable (with a Mean arterial pressure (MAP) <65 mmHg) or died within one hour of ED arrival were excluded. An awake positioning protocol for hypoxemic COVID patients which included changing position every two hours was developed. Oxygen saturation (SpO2) was checked by using bedside pulse oximeter with each step of intervention as defined by the protocol, till the time the patients were either admitted, discharged or referred to other facility. Categorical variables like patients’ age, gender, presentation and duration of symptoms, co-morbid conditions, functional class on arrival and during hospital stay and vital-signs at triage were retrieved from the electronic health record (EHR). All SpO2 readings were recorded with each intervention (nasal cannula, NRM, application of CPAP) and position (sit-up, supine, left lateral, right lateral and prone). The duration of each intervention was noted and entered into a proforma.

Patients who were admitted to the in-patient COVID-Unit were followed and their location (ward/ High dependency unit (HDU)/ Intensive care unit (ICU)) at admission, date and duration of admission, step-up to ICU, invasive ventilation, step-down and final outcome were recorded. Approval was taken from Institutional review board IRB (IRD_IRB_2020_05_001) and all the participants consented to be enrolled in the study.

## RESULTS

A total of 207, COVID-19 positive patients were enrolled in the study with a mean age ± SD of 56.3 ± 13.6 with male predominance (131, 63.3%). Of all patients 188 (90.3%) were in functional class II when they reached the ED, out of which 153 (73.9%) worsened to functional class IV. (Table-I) Hypertension (62.3%) and Diabetes mellitus (54.1%) were found to be the most common comorbidities. Majority of the patients presented with shortness of breath followed by fever and cough (81%, 77.5% and 70% respectively) The disposition included in-patient ICU and HDU admission for 142 (68.6%) patients Table-I. Of the ICU/HDU admissions 130 (91%) did not require invasive ventilation while 12 (9%) went on to be intubated and mechanically ventilated. Non availability of beds resulted in 40 (19.3%) patients referral to other facilities, 7 (3.4%) patients left against medical advice (LAMA), 6 (2.9%) were discharged and 12 (5.8%) expired in the ED. (Table-I) The patients who expired were older than the patients who recovered (Mean age ± SD; 59.8 ± 13.2 versus 52 ± 13.9, p=0.001). Gender distribution was similar in both alive and expired patients (p=0.218). (Table-II).

**Table I:** Patient’s health status at baseline

**Gender; n=207**	
Male	131(63.3)
Female	76(36.7)
**Age**	
Mean ± SD	56.3±13.6
Min-Max	23-85
**Baseline Function class**	
I	1(0.5)
II	188(90.8)
III	17(8.2)
IV	1(0.5)
**Current function class**	
II	15(7.2)
III	39(18.8)
IV	153(73.9)
**Presenting complaints**	
Fever	155(77.5)
Cough	70(35)
Shortness of breath	162(81)
Runny nose	1(0.5)
Sore throat	2(1)
Chest Pain	6(3)
Diarrhea	2(1)
Other complaints	76(38)
**Comorbidities; n=159**	
DM	86(54.1)
HTN	99(62.3)
IHD	18(11.3)
COPD/allergy	13(8.2)
history of TB	4(2.5)
Other	80(50.3)
**Disposition from ED**	
ICU	41(19.8)
Referred out	40(19.3)
Expired in ED	12(5.8)
HDU	101(48.8)
Discharge	6(2.9)
LAMA	7(3.4)

**Table II:** Association of Final outcome with gender, basic functional class, and current functional class.

Final outcome				

	Alive n (%)	Expired n (%)	Total n (%)	p value
**Gender**				
Male	54(62.1)	50(71.4)	104(66.2)	0.218^□^
Female	33(37.9)	20(28.6)	53(33.8)	
Total	87(100)	70(100)	157(100)	
**Basic functional class**				
I	-	1(100)	1(100)	0.132^‡^
II	77(60.6)	50(39.4)	127(100)	
III	4(40)	6(60)	10(100)	
IV	-	1(100)	1(100)	
Total	81(58.3)	58(41.7)	139(100)	
**Current functional class in ED**				
II	10(11.5)	-	10(6.4)	0.002^*‡^
III	19(21.8)	10(14.3)	29(18.5)	
IV	58(66.7)^b^	60(85.7)	118(75.2)	
Total	87(100)	70(100)	167(100)	
**Age**				
Mean ± sd	52±13.9	59.8±13.2	56.3±13.6	0.001^*∫^
Min-Max	23-80	36-84	23-85	

* p value <0.05,

□ Pearson chi-square test,

‡ Fischer exact test,

∫ Independent sample t test.

To improve oxygen saturation with an aim to keep it in the early nineties, a timed position changing protocol was used in all the patients. They were asked to change their position every two hours voluntarily and were allowed to stay in the position of maximum comfort. Since a single patient changed multiple positions, it was seen that majority, 109 (52.7%) had oxygen saturation in nineties in supine position followed by right lateral in 37 (17.9%), while 30 (14.5%) oxygenated maximally while sitting up, 29 (14%) in left lateral position and only 2 (1%) patients got maximum oxygen saturation on prone position. (Table-III) The failure to prone ventilate in our cohort is postulated to be due to the body mass index of more than 25 in 129 (62.3%) of our patients with predominant central obesity.

**Table III:** Frequency of intervention and position for maximum Oxygen saturation.

Interventions provided to patients	N(%)
NRB mask + Nasal Cannula	120(58)
CPAP	81(39.1)
Nasal Cannula	46(22.2)
Room air	34(16.4)
Intubated + Bag	6(2.9)
**Position at which patient reached to maximum oxygen saturation**	
Supine	109(52.7)
Left Lateral	29(14)
Right Lateral	37(17.9)
Sit - up	30(14.5)
Prone	2(1)
**Intervention at which patient reached to maximum oxygen saturation**	
Room air	18(8.7)
Nasal Cannula	36(17.4)
NRB mask + Nasal Cannula	87(42)
CPAP	60(29)
Intubated + Bag	6(2.9)

**Fig.1:**
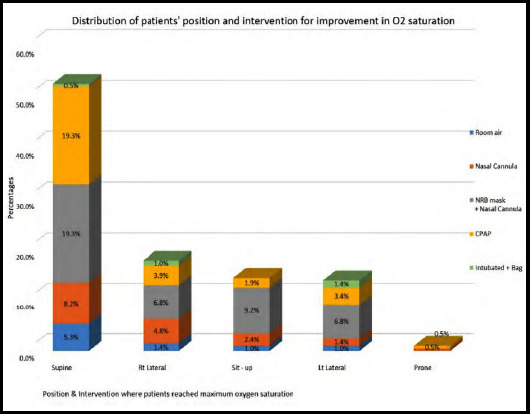


It was observed that out of the 109 patients who preferred to stay in the supine position, the maximal oxygen saturation was obtained in 21 (19%) patients with associated NRM use at 15 liter O2 and nasal prongs use at 5 liters and in those on CPAP. Similar results of maximal oxygenation in the nineties percent were observed with NRM and nasal prongs use in patients kept in the right lateral (6.8%), left lateral (6.8%) and sitting up (9.2%) positions. Therefore, in our study, maximal oxygenation was achieved with NRM and nasal prongs in 87 (42%) of the patients out of 207 patients followed by the use of CPAP in 29 (14%). (Table-III) (Fig.1).

Out of the 142 patients who were admitted to critical care setup (ICU + HDU), 130 did not require invasive ventilation. Of these 73 (56%) had preferred the supine position, 17 (13%) had stayed in the right and left lateral positions and 23 (18%) preferred sitting up. Although it was the favored position in our study population, all the 12 (9%) patients who were intubated later and had to be invasively ventilated, had also preferred to stay in the supine position.

## DISCUSSION

Our study was initially conducted to see the effect of prone positioning on patients with COVID-19, through the use of timed position protocol. However, our patients were not able to tolerate the prone position for more than 15 minutes and predominantly preferred the supine position. This was mostly due to central obesity that has been documented in literature as one of the reasons for failure of prone positioning.^8^ Supine position, right and left lateral and siting up were paired with the use of various modalities of non-invasive ventilation. NRM with supplemental oxygen and application of CPAP was found to work best with supine position. Like other published data, our patients were not able to tolerate prone position with the application of NRM and CPAP, mainly because of lack of beds that can facilitate prone positioning, limited personnel and patient discomfort.^9^ Pressure ulcers and anxiety were other factors that deterred patients from staying prone over prolonged periods.^10^

Covid-19 has been the curve ball no one saw coming. The burden on the healthcare system in terms of preparedness and dealing with the pandemic has been enormous. The effect has been particularly devastating in LMIC where limited resources and lack of established disaster management systems, resulted in catastrophe.^11^ The uncertainty associated with COVID-19 due to lack of previous experience, led to development of many treatment modalities and pathways that have altered over time. This also led to published data with small sample sizes that did not have the required background and insight due to the novelty of the disease and therefore lacked generalizability. A meta-analysis of thirty-five studies (n= 1712 patients) showed improved PaO2/ FiO2 ratio with better SpO2 and lower mortality rates in patients who were prone as compared to those in the supine position.^12^ Many similar studies describing the prone positioning protocol came forth and were well received.^13^ Using the findings of our own study and keeping abreast with the current literature, we hope to extrapolate these results to develop protocols that can be time and cost effective and can improve our patient outcome.

## CONCLUSION

Most of the patients preferred to stay in the supine position and described it as the position of comfort. When used in combination supine position, patients on NRM with nasal prongs and on CPAP, had oxygen saturation in the nineties. Central obesity was found to be the prime reason for the inability to prone our patients. It is our hope that through this cross sectional follow up, we can develop best practice protocols in our ED for patients with COVID-19 in future. This will help us in maximal utilization of our limited resources to improve patient outcome and prevent and/or delay invasive ventilation through a combinations of position change and non-invasive ventilation. This can also lead to generalization of these protocols for limited resource setups and wise use of healthcare resources.

### Authors’ Contribution:

**SA, SGS, AK:** Conceived, designed and edited the manuscript.

**SM & SZ:** Collected data and did the manuscript writing.

**NG:** Did the statistical analysis.
